# WISP1 Predicts Clinical Prognosis and Is Associated With Tumor Purity, Immunocyte Infiltration, and Macrophage M2 Polarization in Pan-Cancer

**DOI:** 10.3389/fgene.2020.00502

**Published:** 2020-05-25

**Authors:** Xia Liao, Yang Bu, Zihan Xu, Fengan Jia, Fan Chang, Junrong Liang, Qingan Jia, Yi Lv

**Affiliations:** ^1^Department of Nutrition, First Affiliated Hospital of Xi’an Jiaotong University, Xi’an, China; ^2^Department of Hepatobiliary Surgery, General Hospital, Ningxia Medical University, Yinchuan, China; ^3^Department of Burns and Plastic Surgery, Shaanxi Provincial People’s Hospital, Xi’an, China; ^4^Metabolite Research Center, Shaanxi Institute of Microbiology, Xi’an, China; ^5^State Key Laboratory of Cancer Biology, National Clinical Research Center for Digestive Diseases and Xijing Hospital of Digestive Diseases, Fourth Military Medical University, Xi’an, China; ^6^Department of Hepatobiliary Surgery, First Affiliated Hospital of Xi’an Jiaotong University, Xi’an, China

**Keywords:** WISP1, tumor purity, macrophage, prognosis, pan-cancer

## Abstract

Cancer is becoming the leading cause of death and a major public health problem. Although many advanced treatment strategies are currently in use, the general prognosis of cancer patients remains dismal due to the high frequency of recurrence, metastasis. The identification of effective biomarkers is important for predicting survival of cancer patients and improving treatment efficacy. In this study, we comprehensively analyzed WNT1-inducible-signaling pathway protein 1 (*WISP1*) expression and explored its correlation with prognosis in pan-cancer using tumor IMmune Estimation Resource (TIMER) and Gene Expression Profiling Interactive Analysis 2 (GEPIA2). We also examined correlations between *WISP1* and immunocyte infiltration using TIMER. We identified genes co-expressed with *WISP1* using the LinkedOmics database and analyzed associated gene ontology using Metascape. Finally, we constructed protein-protein interaction networks and examined correlations between genes co-expressed with *WISP1* and immunocyte infiltration in pan-cancer. *WISP1* level differed between human pan-cancer tissues and normal tissues, indicating its potential as a prognostic biomarker. WISP1 expression was correlated with tumor purity and immunocyte infiltration, especially monocyte-macrophage trafficking and M2 polarization. Genes co-expressed with *WISP1* were mainly associated with extracellular matrix organization, with collagen members *COL6A3*, *COL5A1*, and *COL8A1* being key genes correlated with macrophage infiltration and M2 polarization in pan-cancer. Conversely, in certain types of cancer with better prognoses, *WISP1* was associated with low M2 macrophage infiltration. These results suggest that *WISP1* affect clinical prognosis through associations with tumor purity, immune cell infiltration, and macrophage M2 polarization in pan-cancer, with collagen member proteins may serving as effector molecules of *WISP1*.

## Introduction

According to estimates from the World Health Organization (WHO) in 2015, cancer is the first or second leading cause of death, with increasing incidence and mortality (Bray et al., 2018). In China, cancer is becoming the leading cause of death and a major public health problem (Chen et al., 2016). Although many advanced strategies are used to manage cancer patients, including molecular targeted therapy and immunotherapy, their overall prognosis remains dismal due to high frequency of recurrence, metastasis, and drug resistance.

The CCN protein family in humans comprises six cysteine-rich regulatory proteins that are implicated as multitasking signal integrators in cancer (Yeger and Perbal, 2016). Members of this secreted protein family share a multi-modular structure with an N-terminal secretory signal domain followed by four conserved domains: an insulin-like growth factor binding protein domain, von Willebrand factor type C repeat domain, thrombospondin type I repeat domain, and carboxy-terminal domain (Li et al., 2015). CCN family proteins have highly conserved biological structures, and many studies have described the effects of pathological events in each CCN catalytic domain on cell differentiation (Nishida et al., 2015), migration (Li et al., 2015), mitogenesis (Weiskirchen, 2011), chemotaxis (Zarogoulidis et al., 2015), and angiogenesis (Tsai et al., 2017). However, these proteins have different expression levels and play variable roles in the biology of human cancer (Klenotic et al., 2016). In particular, the expression and function of WNT1-inducible-signaling pathway protein 1 (WISP1) implicates it as an oncogene, but its expression and physiological roles in diverse cancer types remain unclear.

In the present study, we comprehensively analyzed *WISP1* mRNA expression and explored its correlation with prognosis in pan-cancer using Tumor IMmune Estimation Resource (TIMER) and Gene Expression Profiling Interactive Analysis 2 (GEPIA2). We examined the correlation between *WISP1* expression and immunocyte infiltration using TIMER. We examined genes co-expressed with *WISP1* using the LinkedOmics database and analyzed associated gene ontologies (GO) using Metascape. Finally, we constructed protein-protein interaction (PPI) networks and focused on genes co-expressed with *WISP1* to explore its association with immunocyte infiltration in pan-cancer. Our findings suggest that *WISP1* affects clinical prognosis through associations with tumor purity, immune cell infiltration, and macrophage M2 polarization in pan-cancer, with collagen member proteins most likely serving as *WISP1* effector molecules.

## Materials and Methods

### WISP1 Expression and Immune Cell Infiltration in Pan-Cancer

TIMER is a comprehensive resource allowing the systematic analysis of immunocyte infiltrates across diverse cancer types^1^ (Li et al., 2017). Expression levels of *WISP1* in tumor and adjacent normal tissues across different types of cancer were identified across all tumors of The Cancer Genome Atlas (TCGA) via the “Diff Exp” module in TIMER. Correlations between *WISP1* expression and immune cell infiltration, including B cells, CD4^+^ T cells, CD8^+^ T cells, neutrophils, macrophages, and dendritic cells, were explored via “Gene” modules in TIMER with Spearman correlations. Spearman correlations were also calculated to evaluate correlations between *WISP1* expression and markers of monocytes (*CD86*, *CD115*), Tumor-associated macrophages (TAMs; *CCL2*, *CD68*, *IL10*), M1 macrophages (*INOS*, *IRF5*, *COX2*), and M2 macrophages (*CD163*, *VSIG4*, *MS4A4A*), as well as three collagen members (*COL5A1*, *COL6A3*, and *COL8A1*) via “Correlation” modules in TIMER. Correlation outputs were in part graphically presented as heatmaps.

### *WISP1* Expression and Survival Analysis in Pan-Cancer

GEPIA2 is an updated version of GEPIA for analyzing the RNA sequencing data of 9,736 tumor samples and 8,587 normal tissue samples from the TCGA and Genotype-Tissue Expression projects using a standard processing pipeline (Tang et al., 2019). We used GEPIA2 to analyze correlations between *WISP1* expression and survival in various types of cancer. Correlations between *WISP1* expression and markers of immune cell also evaluated using GEPIA2^2^.

The Kaplan-Meier plotter can assess the effects of 54,000 genes on survival in 21 cancer types. Gene expression, overall survival (OS), and recurrence-free survival (RFS) data were downloaded from the Gene Expression Omnibus, European Genome-phenome Archive, and TCGA databases. Correlations between *WISP1* expression and survival in diverse cancer types were analyzed using the Kaplan-Meier plotter^3^ (Nagy et al., 2018). Hazard ratios (HRs) with 95% confidence intervals and log-rank *p*-values were also calculated.

### Genes Co-expressed With *WISP1* in Pan-Cancer

Genes co-expressed with *WISP1* in various cancer types were analyzed using the LinkedOmics database^4^, a publicly available portal that includes multi-omics data from all 32 TCGA cancer types (Vasaikar et al., 2017). The top 50 genes with significant positive correlations with *WISP1* expression in different types of cancer are presented as heatmaps generated by this platform.

### Functional Enrichment Analysis in Pan-Cancer

Metascape is a free, well-maintained, user-friendly gene-list analysis tool for gene annotation and analysis (Zhou et al., 2019) that functions as an automated meta-analysis tool for understanding common and unique pathways within a group of orthogonal, target-discovery studies. In this study, Metascape was used to conduct pathway and process enrichment analysis of the top 100 genes positively correlated with *WISP1* in multiple types of cancer. GO terms for biological processes, cellular components, and molecular function categories, as well as Kyoto Encyclopedia of Genes and Genomes (KEGG) pathways, were enriched using the Metascape online tool^5^. Overlaps between multiple gene lists at the gene and shared term levels were shown in a circos plot. Protein-protein interaction (PPI) enrichment analysis was performed using BioGrid, InWebIM, and OmniPath databases. The Molecular Complex Detection (MCODE) algorithm was applied to identify densely connected network components.

### Data Source and Data Processing

The RNA-seq data (FPKM values) of HNC cohort were downloaded from the Cancer Genome Atlas (TCGA) website^6^. And then, we merged the data using Perl software (mRNA_merge.pl). RNA-seq profiles were normalized using Voom standardized method (variance modeling at the observational level) (Law et al., 2014).

### Quantification of Tumor Cell Immune Cells (TIICs) Using the CIBERSOFT Algorithm

We employed the CIBERSORT method, which is a gene-based deconvolution algorithm that infers 22 human immune cell types and uses the characteristics of 547 marker genes to quantify the relative scores for each cell type, to evaluate the relative proportions of immune cell profiling^7^ (Newman et al., 2015). Total B cells was estimated as a sum of B cells memory and B cells naive. Total CD4 + T cells were calculated as a sum of CD4^+^ naive T cells, CD4^+^ memory resting T cells and CD4^+^ memory activated T cells. Total macrophage fraction was imputed as a sum of M0, M1, and M2 macrophages. And Total B cells was estimated as a sum of B cells memory and B cells naïve. And total dendritic cells were calculated as a sum of dendritic cells resting and dendritic cells activated.

### Statistical Analysis

Survival curves were generated using the Kaplan-Meier plotter. Partial heatmaps were created using GraphPad Prism version 8 (GraphPad Software, La Jolla, CA, United States). Upset plots were created in ImageGP^8^. Gene expression correlations were evaluated using Spearman correlations, and *p* < 0.05 were considered statistically significant.

## Results

### *WISP1* mRNA Levels Differ Between Human Pan-Cancer and the Corresponding Normal Tissues

**FIGURE 1:**
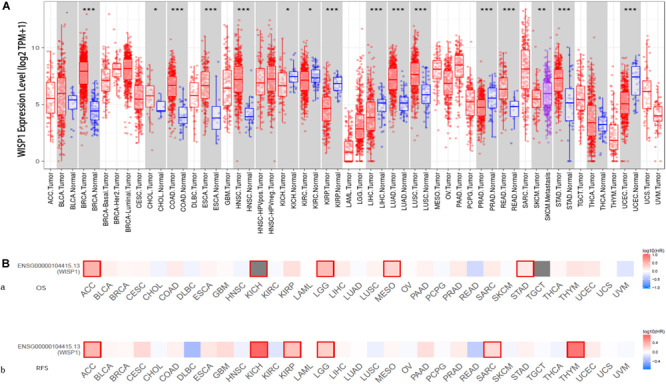
*WISP1* mRNA levels differ between human pan-cancer and normal tissues, suggesting its potential role as a prognostic biomarker. **(A)** *WISP1* mRNA levels were evaluated using TIMER in 33 types of human tumors, and *WISP1* is elevated in most types of human cancer. **(B)** The prognostic impact of WISP1 in 33 types of human tumors were examined using GEPIA2, and high expression of *WISP1* was associated with poor prognosis in most types of human cancer.

The comparison of *WISP1* mRNA levels among diverse cancer types and adjacent normal tissues in TIMER revealed significantly higher *WISP1* expression in breast invasive carcinoma (BRCA), colon adenocarcinoma (COAD), esophageal carcinoma (ESCA), head and neck cancer (HNSC), lung adenocarcinoma (LUAD), lung squamous cell carcinoma (LUSC), rectum adenocarcinoma (READ), and stomach adenocarcinoma (STAD). By contrast, *WISP1* expression was significantly lower than that in adjacent normal tissues in kidney renal papillary cell carcinoma (KIRP), liver hepatocellular carcinoma (LIHC), prostate carcinoma (PRAD), and uterine corpus endometrial carcinoma (UCEC) (Figure 1A).

To evaluate the association between *WISP1* and prognosis, 32 human cancers were investigated using GEPIA2. The relationship between *WISP1* and prognosis varied across different types of cancers. In most cancers, high expression of *WISP1* was associated with shorter OS (Figure 1Ba) and RFS (Figure 1Bb). For further confirmation of this finding, Kaplan-Meier plotter was used to analyse survival in human cancers. Patients in the *WISP1*-high group had shorter OS than those in the *WISP1*-low group for BLCA (HR = 1.79, *p* = 0.0047), KIRP (HR = 2.7, *p* = 0.0006), LIHC (HR = 1.61, *p* = 0.0097), LUSC (HR = 1.39, *p* = 0.0220), OV (HR = 1.58, *p* = 0.0011), PAAD (HR = 1.63, *p* = 0.0300), SARC (HR = 1.91, *p* = 0.0018), STAD (HR = 1.63, *p* = 0.005), and THCA (HR = 1.63, *p* = 0.0300). Patients in the *WISP1*-high group also had shorter RFS than those in the *WISP1*-low group for CESC (HR = 4.28, *p* = 0.0000), KIRP (HR = 3.35, *p* = 0.0180), LUAD (HR = 1.7, *p* = 0.0300), PAAD (HR = 3.5, *p* = 0.0090), SARC (HR = 1.78, *p* = 0.0190), and THCA (HR = 2.14, *p* = 0.0500; Supplementary Figure S1). These findings indicate that *WISP1* expression is elevated in most types of human cancer, indicating that it most likely serve as a biomarker for poor prognosis.

### WISP1 Expression Is Correlated With Tumor Purity and Immunocyte Infiltration in Most Types of Human Cancer

**FIGURE 2:**
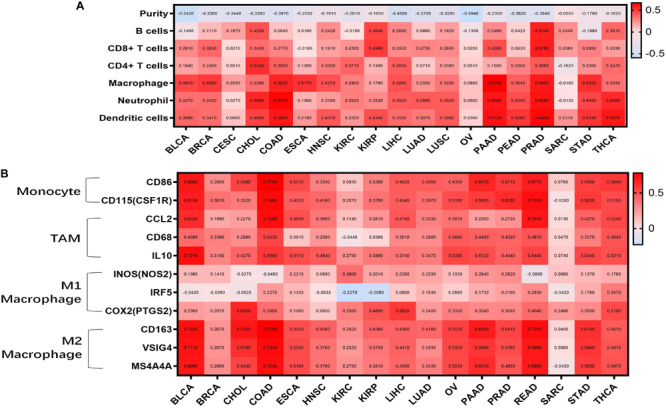
WISP1 expression is correlated with tumor purity, monocyte-macrophage trafficking, and M2 polarization in pan-cancer. **(A)** Correlations between WISP1 expression and infiltrating levels of B cells, CD8^+^ T cells, CD4^+^ T cells, macrophages, neutrophils, and dendritic cells were evaluated in pan-cancer using TIMER. And high expression of *WISP1* is related to low tumor purity with increased levels of immunocyte infiltration in most types of human cancer. **(B)** Correlations between WISP1 and gene markers of macrophage subtype, including monocytes, TAMs, M1, and M2 macrophages, in pan-cancer using TIMER. And high expression of *WISP1* is related to increased levels of M2 macrophage in most types of human cancer.

Tumor-infiltrating lymphocytes are an independent predictor of cancer prognosis (Gilbert et al., 2016). Therefore, we included 19 cancers for which *WISP1* expression was closely related to prognosis to investigate correlations between *WISP1* expression and tumor purity and immunocyte infiltration levels using TIMER. We found negative correlations between *WISP1* expression and tumor purity as well as varying directions and strengths of correlations between *WSIP1* and immunocyte infiltration. *WISP1* expression was significantly correlated with infiltrating levels of B cells in 7 types of cancer, CD8^+^ T cells in 8 types of cancer, CD4^+^ T cells in 9 types of cancer, macrophages in 17 types of cancer, neutrophils in 15 types of cancer, and dendritic cells in 16 types of cancer (Figure 2A). The same correlations between WSIP1 and immunocyte infiltration were confirmed in diverse cancer types using CIBERSORT method (Supplementary Figure S2). These results reveal strong correlations between *WISP1* and immunocyte infiltration, with particularly strong positive correlations with macrophages.

To further investigate correlations between *WISP1* and macrophage subtypes in pan-cancer, we focused on markers of monocytes, TAMs, as well as M1 and M2 macrophages in diverse cancer types using TIMER. *WISP1* expression was positively correlated with infiltrating levels of monocytes in 15 types of cancer, especially BLCA (*CD86*, *r* = 0.6960; *CD115*, *r* = 0.6740), COAD (*CD86*, *r* = 0.7780; *CD115*, *r* = 0.7460), and PAAD (*CD86*, *r* = 0.6470; *CD115*, *r* = 0.5660); with infiltrating levels of TAMs in 15 types of cancer, especially BLCA (*CCL2*, *r* = 0.5320; *CD68*, *r* = 0.4080; *IL10*, *r* = 0.7270), COAD (*CCL2*, *r* = 0.7270; *CD68*, *r* = 0.5420; *IL10*, *r* = 0.5860), and READ (*CCL2*, *r* = 0.7010; *CD68*, *r* = 0.4810; *IL10*, *r* = 0.5440); and with infiltrating levels of M2 macrophages in 16 types of cancer, especially BLCA (*CD163*, *r* = 0.7220, *VSIG4*, *r* = 0.7110, *MS4A4A r* = 0.6990), COAD (*CD163*, *r* = 0.7790, *VSIG4*, *r* = 0.7440, *MS4A4A*, *r* = 0.7240), and PAAD (*CD163*, *r* = 0.5850, *VSIG4*, *r* = 0.5980, *MS4A4A, r* = 0.5310). Correlations between *WISP1* expression and M1 macrophages were weak or non-existent (Figure 2B and Supplementary Figure S3). These findings indicate that high expression of *WISP1* is related to low tumor purity with increased levels of trafficking monocytes and M2 macrophage polarization in most types of human cancer.

### Genes Co-expressed With WISP1 Are Mainly Associated With ECM Organization

**FIGURE 3:**
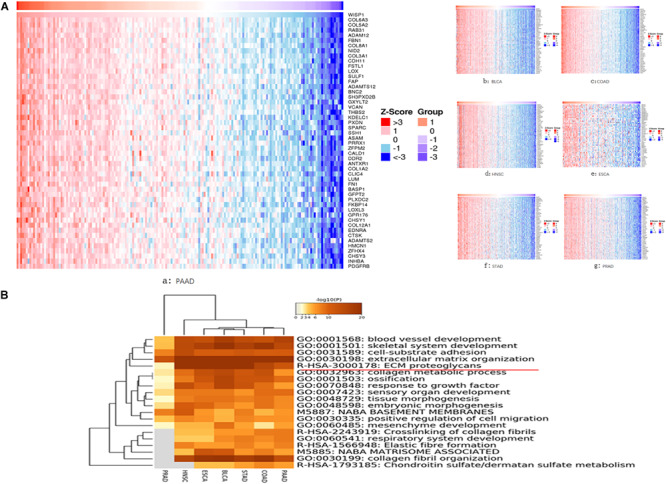
Genes co-expressed with WISP1 were examined and their enriched terms were mainly in ECM organization. **(A)** Genes co-expressed with WISP1 in seven types of cancer (BLCA, ESCA, HNSC, PRAD, COAD, PAAD, and STAD) were examined using the LinkedOmics database. And *COL6A3* and its family collagen members appeared most frequently. **(B)** Genes co-expressed with WISP1 were significantly enriched in ECM organization in the selected seven cancers explored using Metascape.

We further investigated seven types of cancer (BLCA, ESCA, HNSC, PRAD, COAD, PAAD, and STAD) in which *WSIP1* expression was closely related to tumor purity and immunocyte infiltration. To identify genes correlated with *WISP1* expression in these cancers, we performed systematic analysis using the LinkedOmics database, which compiles data on co-expression. In PAAD, the top 10 related genes were *COL6A3, COL5A2, RAB31, ADAM12, FBN1, COL8A1, NID2, COL3A1, CDH11*, and *FSTL1*. In the other six cancers, *COL6A3* and its family collagen members appeared most frequently, all of which were associated with extracellular matrix (ECM) organization in GO analysis (Figure 3A).

We further explored the top 100 genes co-expressed with *WISP1* in the selected seven cancers using Metascape. Accumulative hypergeometric *p*-values were calculated and shown as a heatmap for the top 20 clusters with enriched GO/KEGG terms. Genes co-expressed with *WISP1* were significantly enriched in ECM organization in seven cancers (Figure 3B). Co-expressed genes were also significantly enriched in collagen fibril organization in six cancers (PRAD data missing; Figure 3B). These results suggest that genes co-expressed with *WISP1* may act as tumor promotors by regulating ECM and collagen fibril organization in most types of human cancer.

### Collagen Members Are Associated With ECM Organization in Pan-Cancer

**FIGURE 4:**
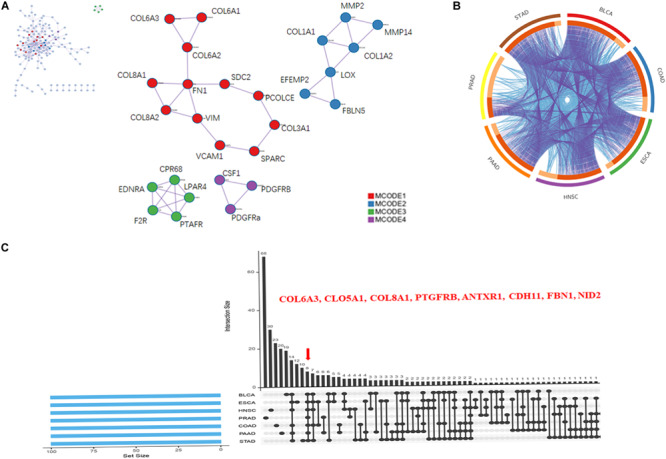
Collagen members *COL6A3*, *COL5A1*, and *COL8A1* are key coincident genes of WISP1 and are correlated with ECM organization. **(A)** A PPI network of the top 100 genes co-expressed with WISP1 in the selected seven cancers was constructed using Cytoscape. **(B)** Overlaps among the top 100 genes co-expressed with WISP1 in the selected seven cancers are shown in a circos plot. **(C)** Gene overlap was also visualized in the selected seven cancers using ImageGP.

PPIs are crucial cellular mechanisms that reveal information on protein co-expression and co-localization, genetic interactions, intersecting pathways, and physical molecular interactions. In the present study, we extracted all PPIs within each input gene list from a PPI data source to form a PPI network visualized using Cytoscape. Next, GO enrichment and MCODE network analysis were applied to the network. Each MCODE network was assigned a unique color. This analysis showed that networks involving the selected genes were mainly related to ECM organization, with significant interactions among the collagen members *COL6A3, COL5A1, and COL8A1* (Figure 4A).

We next generated a circos plot showing overlaps between genes based on their functions or shared pathways. Larger numbers of purple links and longer dark orange arcs indicate greater overlap among the input gene lists, and blue links indicate functional overlap among the input gene lists (Figure 4B). We further visualized overlaps among 100 genes for each of the seven selected cancers using ImageGP. We observed eight overlapping genes: *COL6A3, COL5A1, COL8A1, PTGFRB, ANTXR1, CDH11, FBN1, and NID2* (Figure 4C). Together, these results suggest that the collagen members *COL6A3*, *COL5A1*, and *COL8A1* are key genes in the seven selected cancers, reflecting alterations in ECM organization.

### Genes Co-expressed With WISP1 Are Correlated With Macrophage Infiltration and M2 Polarization in Diverse Cancer Types

**FIGURE 5:**
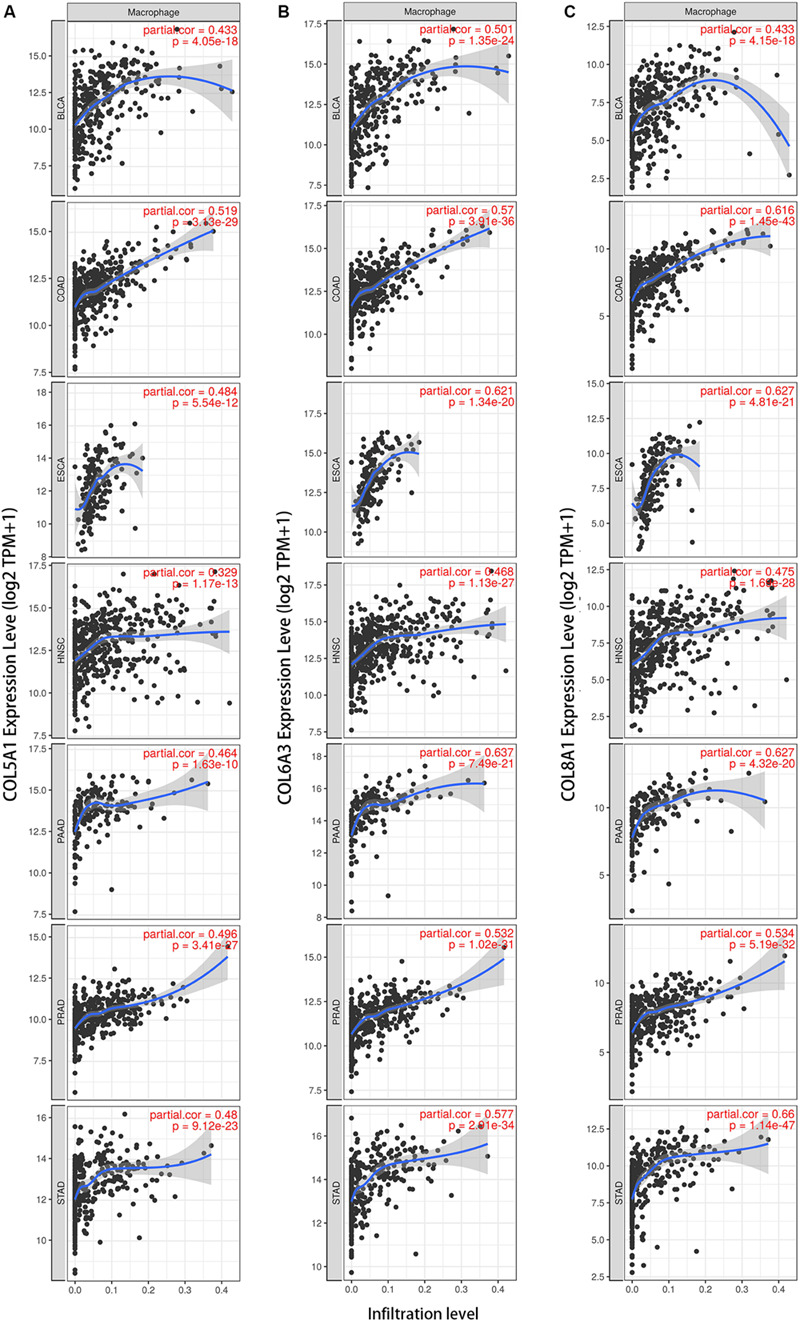
Collagen members *COL6A3*, *COL5A1*, and *COL8A1* are positively correlated with the infiltrating levels of macrophage. Expression of *COL5A1* **(A)**, *COL6A3* **(B)**, and *COL8A1* **(C)** were positively correlated with infiltrating levels of macrophages in the selected seven cancers using TIMER.

We next examined correlations between expression of the collagen members *COL6A3, COL5A1, and COL8A1* and markers of macrophages in the seven selected cancer types in TIMER. In a majority of cancers, expression of were positively correlated with infiltrating levels of macrophages *COL5A1* (BLCA, *r* = 0.674; COAD, *r* = 0.519; ESCA, *r* = 0.484; HNSC, *r* = 0.329; PAAD, *r* = 0.464; PRAD, *r* = 0.496; STAD, *r* = 0.480; Figure 5A), COL6A3 (BLCA, *r* = 0.501; COAD, *r* = 0.57; ESCA, *r* = 0.621; HNSC, *r* = 0.466; PAAD, *r* = 0.637; PRAD, *r* = 0.532; STAD, *r* = 0.577; Figure 5B), and *COL8A1* (BLCA, *r* = 0.433; COAD, *r* = 0.616; ESCA, *r* = 0.621; HNSC, *r* = 0.468; PAAD, *r* = 0.627; PRAD, *r* = 0.534; STAD, *r* = 0.66; Figure 5C). And the positively correlation between the infiltrating levels of macrophages and the expression of COL5A1, COL6A3, and COL8A1 were confirmed in diverse cancer types using CIBERSORT method (Supplementary Figure S4).

**FIGURE 6:**
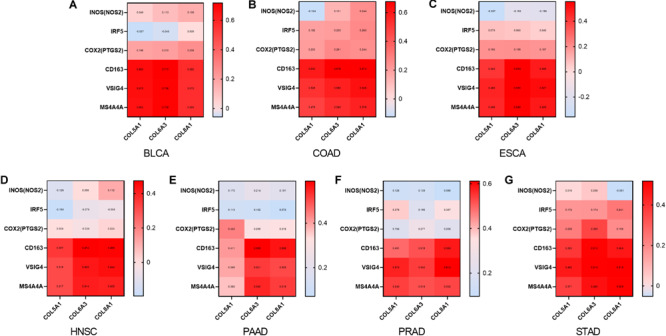
Collagen members *COL6A3*, *COL5A1*, and *COL8A1* are positively correlated with M2 macrophage. Correlations between *COL6A3*, *COL5A1*, *COL8A1* expression and levels of M1 and M2 macrophage in **(A)** BLCA, **(B)** COAD, **(C)** ESCA, **(D)** HNSC, **(E)** PAAD, **(F)** PRAD, and **(G)** STAD using GEPIA2.

We also examined correlations between expression of *COL6A3, COL5A1*, and *COL8A1* with M1 and M2 macrophages in the seven selected types of cancer. *COL6A3, COL5A1*, and *COL8A1* expression were most highly correlated with *CD163, VSIG4*, and *MS4A4A* of the M2 phenotype (Figure 6). By contrast, *COL6A3, COL5A1*, and *COL8A1* expression were less correlated with *NOS2, IRF5, PTGS2* of the M1 phenotype. These findings suggest that genes co-expressed with *WISP1* including *COL6A3, COL5A1*, and *COL8A1* are correlated with macrophage M2 polarization.

### *WISP1* Expression Is Associated With Low Immunocyte Infiltration and Absence of Macrophage M2 Polarization in Tumors With Better Prognoses

**FIGURE 7:**
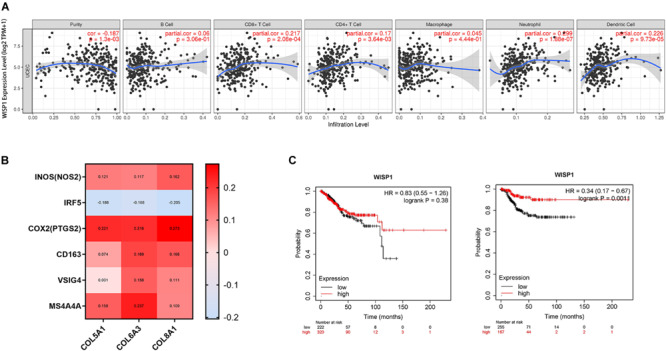
WISP1 expression with low immunocyte infiltration and no macrophage M2 polarization is associated with better UCEC prognosis. **(A)** WISP1 expression was weakly or not correlated with tumor purity or macrophages in UCEC using TIMER. **(B)** The M1 phenotype of macrophages was more highly correlated with WISP1 expression than the M2 phenotype in UCEC using GEPIA2. **(C)** Kaplan-Meier plotter analysis showed that UCEC patients in the WISP1-high group had longer OS and RFS than those in the WISP1-low group.

UCEC, which has a better prognosis, was used to investigate correlations between *WISP1* expression and immunocyte infiltration in TIMER. *WISP1* expression was weakly correlated with tumor purity (*r* = -0.187, *p* = 0.0013), not correlated with infiltrating levels of B cells (*r* = 0.06, *p* = 0.3060), and weakly correlated with CD8^+^ T cells (*r* = 0.217, *p* = 0.0002), CD4^+^ T cells (*r* = 0.17, *p* = 0.0036), macrophages (*r* = 0.045, *p* = 0.4440), neutrophils (*r* = 0.299, *p* = 0.0000), and dendritic cells (*r* = 0.266, *p* = 0.0000; Figure 7A). And the correlations were also reconfirmed using CIBERSORT method (Supplementary Figure S5). When we examined correlations between *WISP1* expression and markers of M1 and M2 macrophages, we found that *WISP1* expression was more highly correlated with the M1 phenotype of macrophages (Figure 7B). Furthermore, using Kaplan-Meier plotter to analyse survival, UCEC patients in the *WISP1*-high group had better prognosis with longer RFS (HR = 0.35, *p* = 0.0051) than those in the *WISP1*-low group (Figure 7C). These findings suggest that *WISP1* expression without macrophage infiltration or M2 polarization is associated with better prognoses in UCEC.

## Discussion

Solid tumors are complex entities because they are surrounded by a heterogeneous array of ECM and various stromal cells (Quail and Joyce, 2013). A series of genetic and phenotypic changes in cancer cells and infiltration of a diversity of immunocytes in the tumor microenvironment can orchestrate the process of malignant transformation into a permissive state that promotes tumor progression (Sullivan et al., 2019). Although cancer immunotherapies show anti-tumor efficacy for some types of solid tumors, tumor cells employ camouflage and evolve to evade immune system attacks. Thus, the identification of effective biomarkers is important for predicting survival of cancer patients and improving treatment efficacy.

In human cancers, levels of CCN protein expression are closely correlated with some biological characteristics and clinical features, including venous invasion, cellular differentiation, TNM stage, RFS, and OS (Jia et al., 2016). Although the expression and function of WISP1 implicates it as an oncogene, it may play diverse roles in different types of cancer. In prostate adenocarcinoma, WISP1 expression is associated with shorter biochemical RFS (Gaudreau et al., 2019). In melanoma, the presence of WISP1 in the tumor microenvironment stimulates invasion and metastasis by promoting an epithelial-mesenchymal transition-like process (Deng et al., 2019). By contrast, lower levels of WISP1 expression are observed in breast cancer patients with poor prognoses, suggesting that WISP1 acts as a tumor suppressor in breast cancer (Davies et al., 2007). WISP1 overexpression in lung cancer cells leads to inhibition of *in vitro* cell invasion and motility as well as lung metastasis (Soon et al., 2003). Thus, dysregulation of WISP1 signaling has pathological effects, and WISP1 has varying physiological roles in different types of cancer. Therefore, extensive investigation of WISP1 is necessary to determine its involvement in different cancer types.

In the present study, we showed that WISP1 expression differs between tumor tissues and adjacent normal tissues, suggesting its role as a prognostic biomarker depending on the type of cancer. *In vitro*, WISP1 could induce epithelial-mesenchymal transition in tumor cells, leading to increased migration and invasion, however, *in vivo*, WISP1 could play different roles in diverse cancer types (Ono et al., 2013; Gurbuz and Chiquet-Ehrismann, 2015; Wu et al., 2016). This raises debate as to whether the malignant phenotype of tumors is largely determined by the host genetic background or the immune status of the tumor microenvironment. Matricellular proteins, including those of the CCN family, are dynamically secreted and exert regulatory rather than structural functions in the ECM, but they are often dysregulated in pathological processes (Kim et al., 2018). Therefore, it may be more meaningful to analyse the regulatory role of ECM genes in the context of the immune microenvironment. Our present results show that expression of WISP1 is negatively correlated with tumor purity and has differing strengths of correlations with immunocyte infiltration. In most cancers, WISP1 expression had strong positive correlations with M2 macrophages. M1 macrophages, however, were weakly or not correlated with WISP1 expression. In established tumors, monocytes are constantly trafficked into the tumor tissue where they develop into M2 macrophages and achieve tumor immune evasion (Yang et al., 2018). Thus, our findings suggest that WISP1 influences clinical prognoses through its associations with tumor purity, immune infiltration, and macrophage M2 polarization in most types of human cancer.

To further analyse the role of WISP1 in regulating the immune microenvironment, the top 100 genes co-expressed with WISP1 were examined in seven selected cancers using the LinkedOmics database, and enriched GO/KEGG terms were explored using Metascape. We showed that genes co-expressed with WISP1 may act as tumor promotors by regulating ECM organization, with the collagen members *COL6A3*, *COL5A1*, and *COL8A1* being key genes in a majority of cancers. Collagens, which are major components of the ECM, are involved in the regulation of tumor cell proliferation, migration, and invasion. Collagen members, including COL6A3, COL5A1, and COL8A1, act as oncogenes in the progression of different types of cancer and are highly correlated with poor prognosis in cervical cancer patients (Liu et al., 2018; Shang et al., 2018; Feng et al., 2019). However, the relationship between collagen members and the immune microenvironment is still unclear, with no studies showing a correlation between macrophage infiltration and M2 polarization. In this study, we showed that COL6A3, COL5A1, and COL8A1 most likely be effector molecules of WISP1 and were positively correlated with monocyte infiltration and M2 polarization in pan-cancer.

## Conclusion

High expression of WISP1 is associated with poor prognoses in various cancer types; WISP1 could serve as a prognostic biomarker when considering tumor purity and macrophage infiltration. Also, enhanced monocyte-macrophage trafficking and M2 polarization are correlated with expression of collagen members COL6A3, COL5A1, and COL8A1, which may orchestrate the cancer-promoting effects of WISP1. These findings shed light on the pro-carcinogenic role of WISP1 in pan-cancer, suggesting that WISP1 affects clinical prognosis through associations with tumor purity, immune infiltration, and macrophage M2 polarization, and collagen member proteins may serving as effector molecules.

## Data Availability Statement

Publicly available datasets were analyzed in this study. This data can be found here: https://portal.gdc.cancer.gov/.

## Author Contributions

XL, YB, ZX, FJ, FC, JL, QJ, and YL contributed to the study design and analyses. QJ conceived the study. XL and YB performed the majority of the bioinformatics analyses. ZX, FJ, FC, and JL participated in data analysis and visualization. QJ and YL drafted and prepared the manuscript. All authors approved the final manuscript.

## Conflict of Interest

The authors declare that the research was conducted in the absence of any commercial or financial relationships that could be construed as a potential conflict of interest.

## Footnotes

1 https://cistrome.shinyapps.io/timer/

2 http://gepia2.cancerpku.cn/#index

3 http://kmplot.com/analysis/

4 http://www.linkedomics.org

5 http://metascape.org

6 https://cancergenome.nih.gov/

7 https://cibersort.stanford.edu/index.php

8 http://www.ehbio.com/ImageGP/index.php/Home/Index/index.html

## Supplementary Material

FIGURE S1 High expression of *WISP1* is a prognostic biomarker predicting shorter OS and RFS in most types of human cancer.

FIGURE S2 Correlation of *WISP1* expression with immune infiltration level in diverse cancer types. WISP1 expression is significantly negatively related to tumor purity and has positive correlations with infiltrating levels of macrophages in various cancer types evaluated by CIBERSORT method.

FIGURE S3 Correlation analysis between *WISP1* and gene markers of monocytes and macrophages in GEPIA2. WISP1 expression was positively correlated with markers of monocytes. Gene markers of M1 macrophages showed weak correlations with *WISP1* expression, whereas M2 macrophage markers showed moderate and strong correlations.

FIGURE S4 Collagen members *COL6A3*, *COL5A1*, and *COL8A1* were correlated with macrophage infiltration in various cancer types. Expression of *COL5A1*, *COL6A3*, and *COL8A1* were positively correlated with infiltrating levels of macrophages in diverse cancer types using CIBERSORT method.

FIGURE S5 Correlation of *WISP1* expression with immune infiltration level in UCEC. *WISP1* expression has no significant correlations with infiltrating levels of B cells, CD8^+^ T cells, CD4^+^ T cell, macrophages, neutrophils, and dendritic cells in UCEC.
