# Supplementary figures and images for: WISP1 Predicts Clinical Prognosis and Is Associated With Tumor Purity, Immunocyte Infiltration, and Macrophage M2 Polarization in Pan-Cancer
Source: Front Genet. 2020 May 25;11:502. doi: 10.3389/fgene.2020.00502 (PMC7261883; doi:10.3389/fgene.2020.00502)

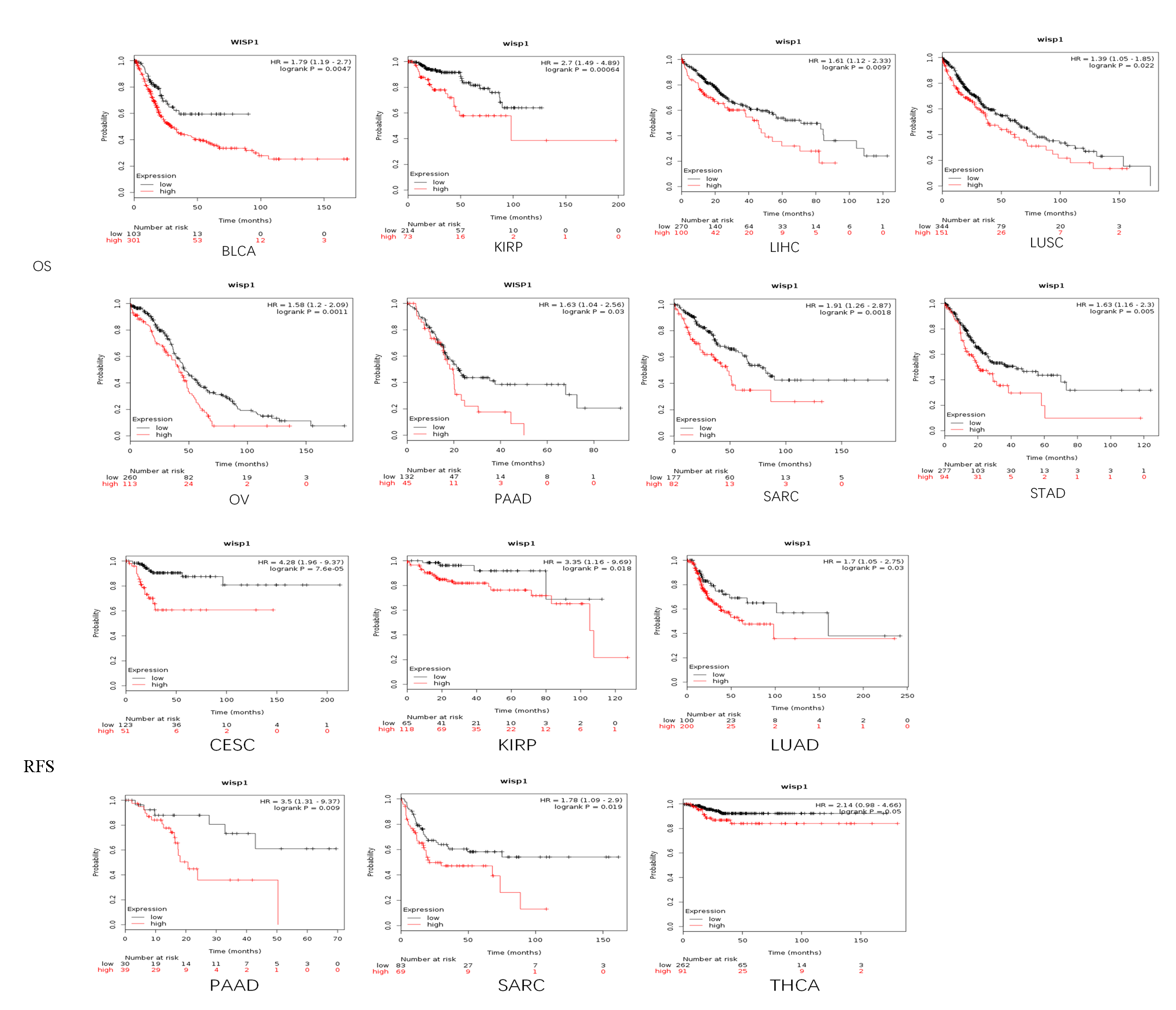

Supplement: FIGURE S1 — High expression of WISP1 is a prognostic biomarker predicting shorter OS and RFS in most types of human cancer. [file Image_1.TIF]

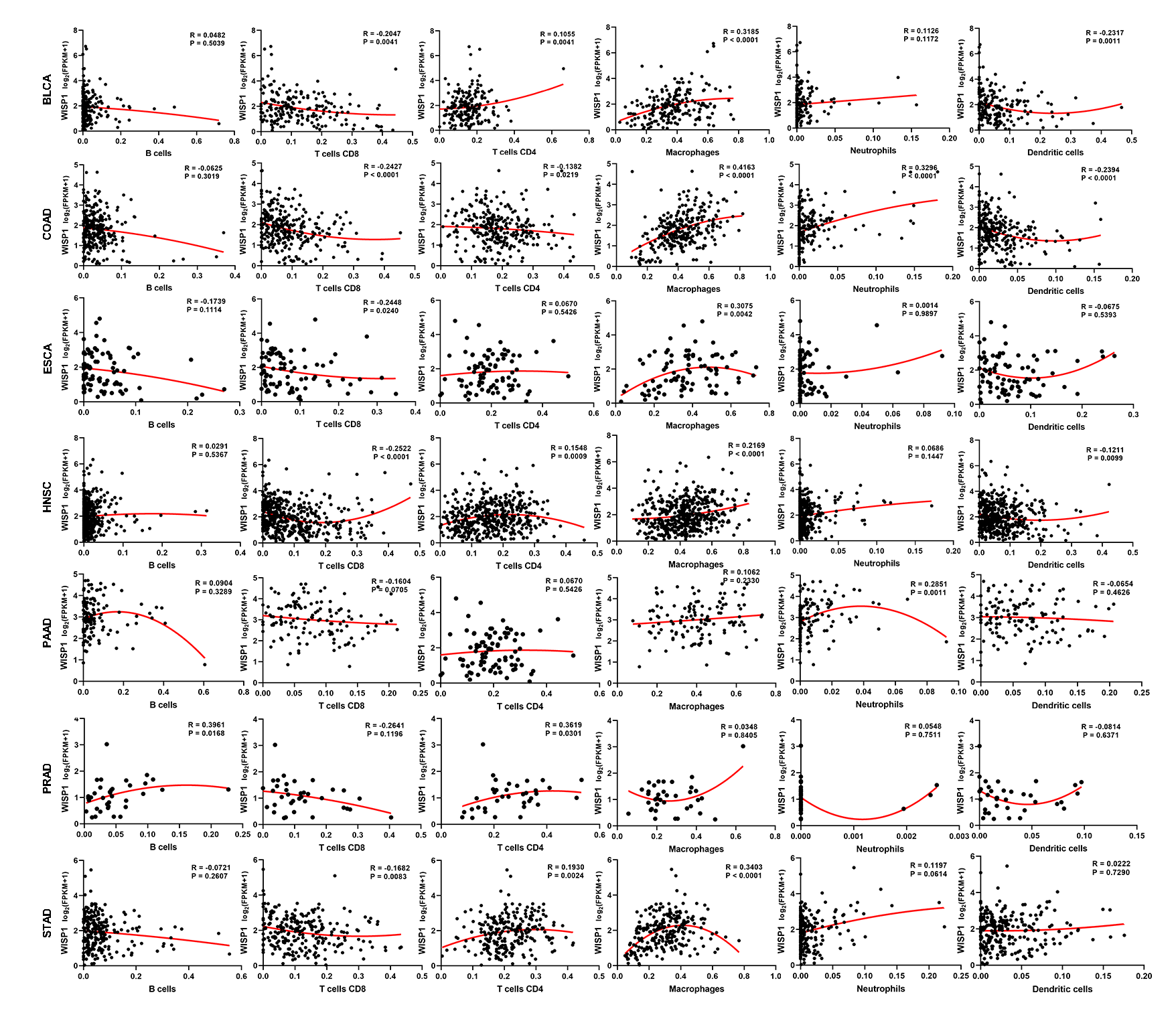

Supplement: FIGURE S2 — Correlation of WISP1 expression with immune infiltration level in diverse cancer types. WISP1 expression is significantly negatively related to tumor purity and has positive correlations with infiltrating levels of macrophages in various cancer types evaluated by CIBERSORT method. [file Image_2.TIF]

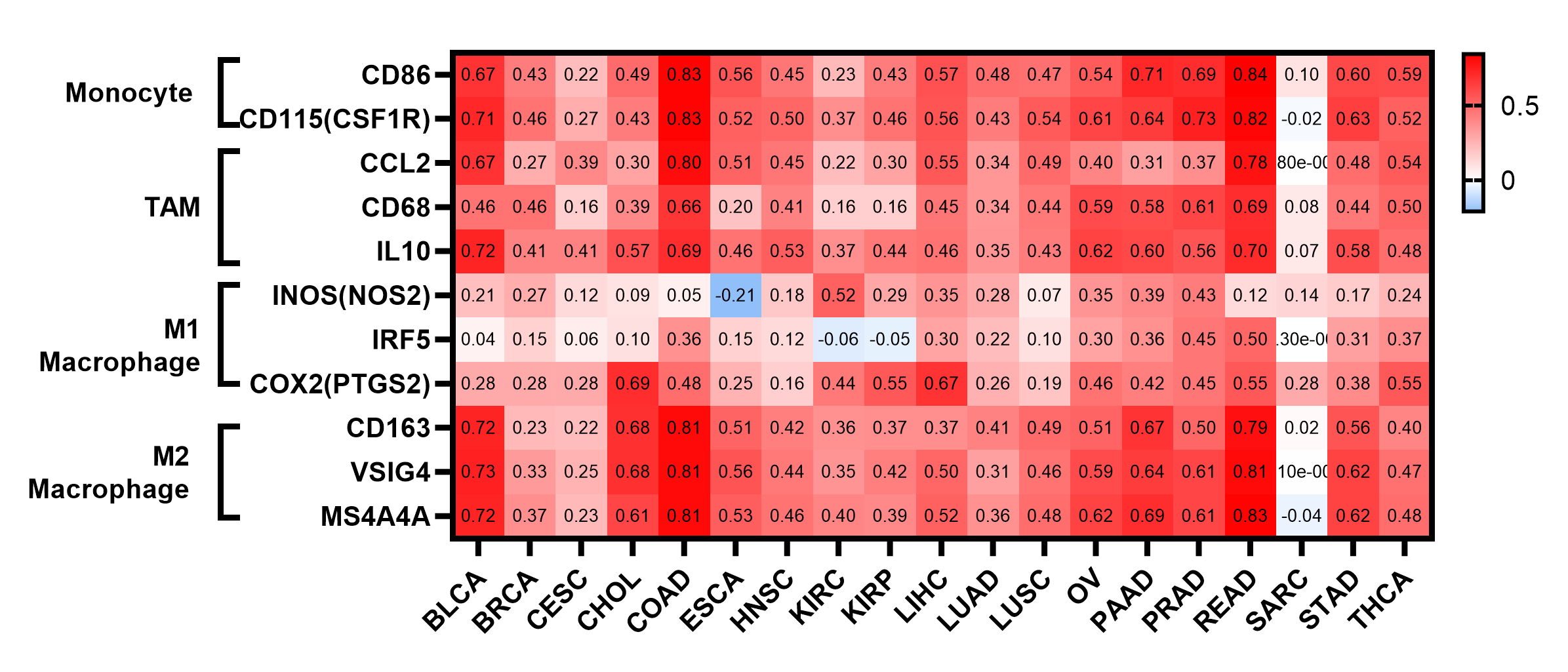

Supplement: FIGURE S3 — Correlation analysis between WISP1 and gene markers of monocytes and macrophages in GEPIA2. WISP1 expression was positively correlated with markers of monocytes. Gene markers of M1 macrophages showed weak correlations with WISP1 expression, whereas M2 macrophage markers showed moderate and strong correlations. [file Image_3.TIF]

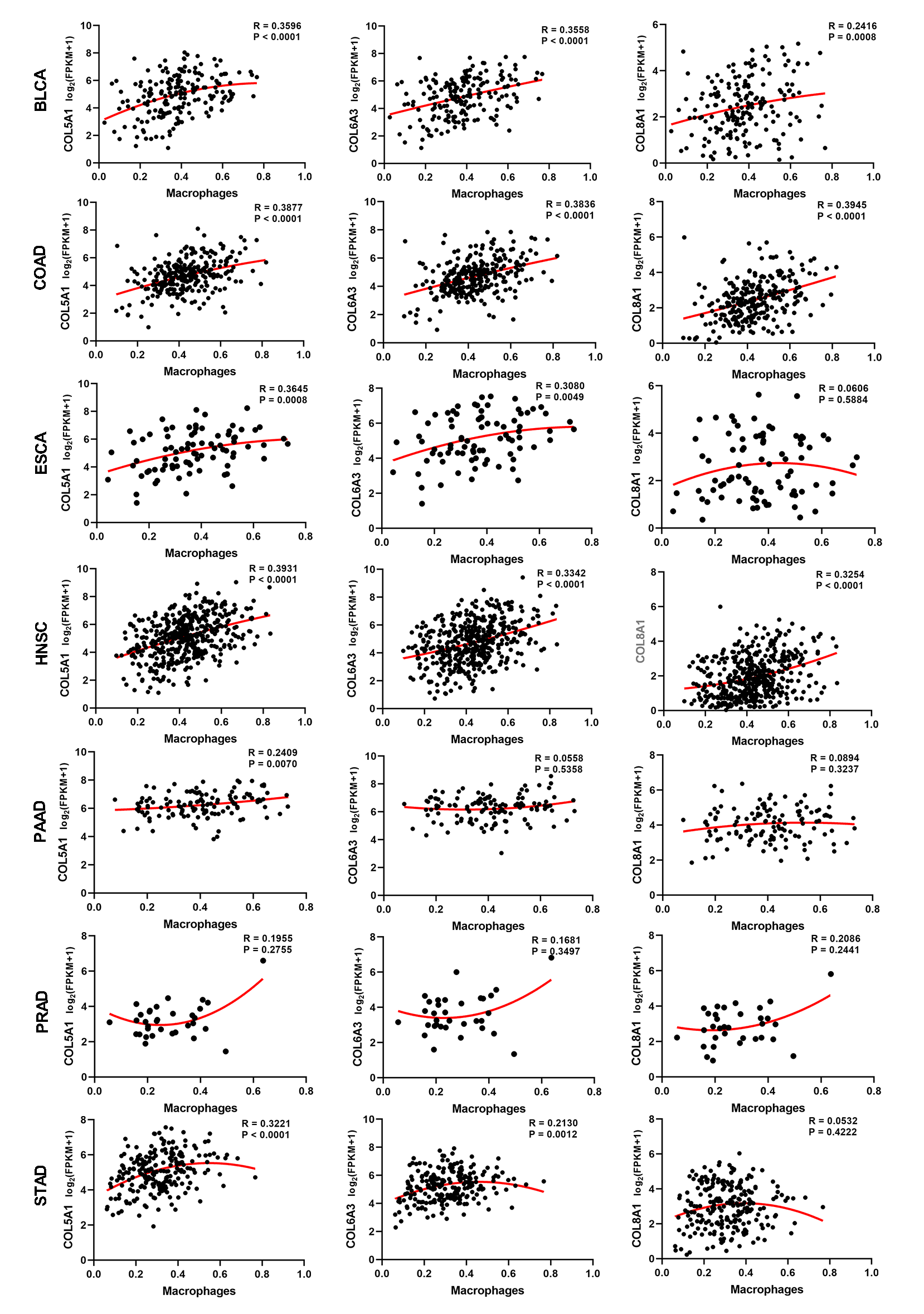

Supplement: FIGURE S4 — Collagen members COL6A3, COL5A1, and COL8A1 were correlated with macrophage infiltration in various cancer types. Expression of COL5A1, COL6A3, and COL8A1 were positively correlated with infiltrating levels of macrophages in diverse cancer types using CIBERSORT method. [file Image_4.TIF]

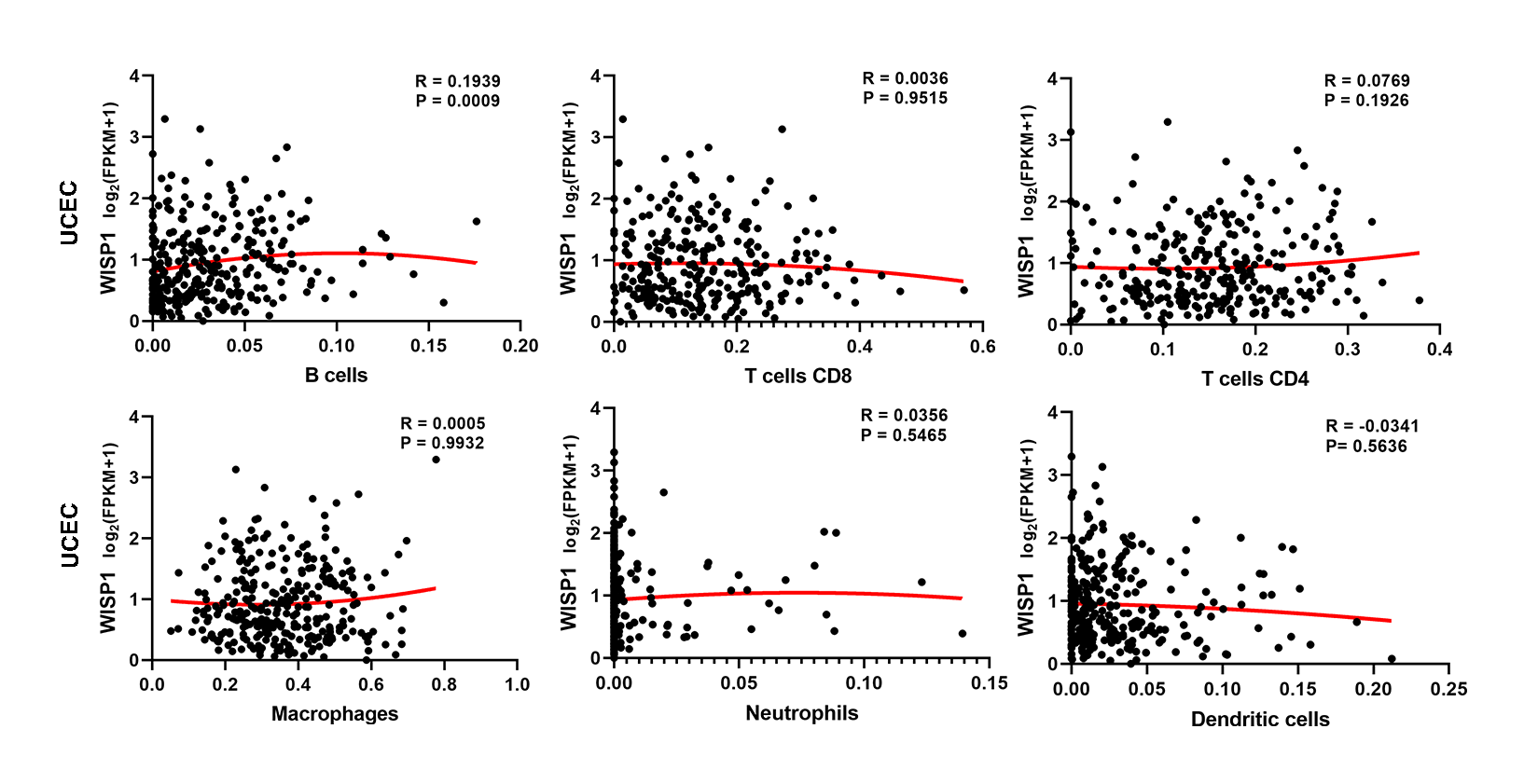

Supplement: FIGURE S5 — Correlation of WISP1 expression with immune infiltration level in UCEC. WISP1 expression has no significant correlations with infiltrating levels of B cells, CD8+ T cells, CD4+ T cell, macrophages, neutrophils, and dendritic cells in UCEC. [file Image_5.TIF]
